# Mechanisms of Triptolide-Induced Hepatotoxicity and Protective Effect of Combined Use of Isoliquiritigenin: Possible Roles of Nrf2 and Hepatic Transporters

**DOI:** 10.3389/fphar.2018.00226

**Published:** 2018-03-16

**Authors:** Zhenyan Hou, Lei Chen, Pingfei Fang, Hualin Cai, Huaibo Tang, Yongbo Peng, Yang Deng, Lingjuan Cao, Huande Li, Bikui Zhang, Miao Yan

**Affiliations:** ^1^Department of Pharmacy, The Second Xiangya Hospital, Central South University, Changsha, China; ^2^Institute of Clinical Pharmacy, Central South University, Changsha, China; ^3^Department of Pharmacy, Chemistry College, Xiangtan University, Xiangtan, China; ^4^Molecular Science and Biomedicine Laboratory, College of Life Sciences, State Key Laboratory of Chemo, Bio-Sensing and Chemometrics, Hunan University, Changsha, China; ^5^School of Pharmacy, Hunan University of Chinese Medicine, Changsha, China

**Keywords:** triptolide, hepatotoxicity, isoliquiritigenin, nuclear transcription factor E2-related factor 2, transporter, bile acid

## Abstract

Triptolide (TP), the main bioactive component of *Tripterygium wilfordii* Hook F, can cause severe hepatotoxicity. Isoliquiritigenin (ISL) has been reported to be able to protect against TP-induced liver injury, but the mechanisms are not fully elucidated. This study aims to explore the role of nuclear transcription factor E2-related factor 2 (Nrf2) and hepatic transporters in TP-induced hepatotoxicity and the reversal protective effect of ISL. TP treatment caused both cytotoxicity in L02 hepatocytes and acute liver injury in mice. Particularly, TP led to the disorder of bile acid (BA) profiles in mice livers. Combined treatment of TP with ISL effectively alleviated TP-induced hepatotoxicity. Furthermore, ISL pretreatment enhanced Nrf2 expressions and nuclear accumulations and its downstream NAD(P)H: quinine oxidoreductase 1 (NQO1) expression. Expressions of hepatic P-gp, MRP2, MRP4, bile salt export pump, and OATP2 were also induced. In addition, *in vitro* transport assays identified that neither was TP exported by MRP2, OATP1B1, or OATP1B3, nor did TP influence the transport activities of P-gp or MRP2. All these results indicate that ISL may reduce the hepatic oxidative stress and hepatic accumulations of both endogenous BAs and exogenous TP as well as its metabolites by enhancing the expressions of Nrf2, NQO1, and hepatic influx and efflux transporters. Effects of TP on hepatic transporters are mainly at the transcriptional levels, and changes of hepatic BA profiles are very important in the mechanisms of TP-induced hepatotoxicity.

## Introduction

Triptolide, the main bioactive component of the traditional Chinese herb TwHF, possesses various pharmacological properties (Wang et al., 2004; Liu, 2011; Li X. et al., 2014). However, the clinical application of TP is limited due to its toxicities, especially its high incidence of hepatotoxicity and serious consequences.

With regard to the mechanisms of TP-induced hepatotoxicity, previous studies have indicated that oxidative stress damage induced by TP is important (Wang J. et al., 2013), which is shown by increased ROS levels and decreased content of GSH in the liver (Li J. et al., 2014; Li X. et al., 2014). Besides, since TP-induced hepatotoxicity is quantitatively correlated with the hepatic exposure to the parent drug, changes in pharmacokinetic profiles of TP may also influence its hepatotoxicity (Kong et al., 2015), and CYP3A-mediated metabolism of TP is a detoxification pathway (Xue et al., 2011). More recently, TP administration was found to increase the total bile acid (TBA) accumulation in rat livers (Jiang et al., 2016; Yang et al., 2017), and activation of the BA receptor (FXR) ameliorates the TP-induced liver injuries (Jin et al., 2015). Combined with the cholestatic symptoms of TP administration observed in clinics (Xue et al., 2009), we speculate that besides the hepatic accumulation of TP, mechanisms involved in the change of endogenous BA profiles may as well contribute to TP-induced hepatotoxicity.

Hepatic influx and efflux transporters localized to the canalicular or basolateral membrane of hepatocytes are responsible for the movement of endogenous and exogenous compounds (e.g., BAs, drugs, and metabolites) across the cellular membranes, thus playing a critical role in determining the drug toxicity and efficacy (Klaassen and Aleksunes, 2010; Corsini and Bortolini, 2013). Changes in either expressions or activities of transporters contribute to the variability in drug hepatic exposure as well as the hepatic endogenous chemical accumulation and BA-dependent or -independent bile flows (Hillgren et al., 2013; Pfeifer et al., 2014). Since TP is predominantly metabolized in the liver and mainly secreted in the bile (Liu et al., 2014), hepatic transporters play an important role in modulating TP-induced hepatotoxicity and may serve as the treatment target. It has been proved that TP is the substrate of P-gp (ABCB1, MDR1) rather than a BCRP (ABCG2) (Zhuang et al., 2013). Inhibition of the expression and function of P-gp augments the TP-induced liver injuries (Kong et al., 2015). However, apart from P-gp, whether other hepatic transporters participate in TP transport and further hepatotoxicity has not been investigated, such as the efflux transporters MRPs (ABCCs) and the influx transporters OATPs, both of which have wide substrate specificity and are identified to be very important in clinical practice (Hillgren et al., 2013). On the other hand, whether TP causes disorders of hepatic BA profiles by modulating the BA transporter expressions or activities has not been made clear.

Nuclear receptors are transcription factors involved in the maintenance of BA homeostasis and drug disposition by regulating phase I and phase II metabolism as well as phase 0 and phase III transports (Zollner et al., 2010). Among them, Nrf2 is the primary player in the cell defense system and may activate the expressions of antioxidant enzymes, detoxification enzymes, and transporters such as MRPs and BSEP (ABCB11) (Klaassen and Reisman, 2010), promoting the antioxidation, detoxification, and elimination of harmful xenobiotics and BA homeostasis, thus protecting against the DILI (Weerachayaphorn et al., 2012; Kamisako et al., 2014; Zhang et al., 2017). Activation of Nrf2 can protect against TP-induced hepatotoxicity by combating the oxidative stress (Li J. et al., 2014), indicating that activation of Nrf2 and its downstream protective genes may be effective in preventing TP-induced liver injuries.

Licorice (*Glycyrrhiza uralensis*) is usually used as a unique “guide drug” in China to improve the efficacy and reduce the toxicity of other ingredients (Wang X. et al., 2013). Its main bioactive compounds have been used in combination with TwHF/TP to reduce the hepatotoxicity in clinical practice (Li X. et al., 2014). Our previous studies have demonstrated that ISL is the most potent Nrf2 inducer among the four compounds derived from licorice and can activate Nrf2 downstream cytoprotective enzymes and transporters (Gong et al., 2015). We also show that ISL may protect against TP-induced oxidative stress in HepG2 cells and mouse livers via Nrf2 activation (Cao et al., 2016a,b). Based on our previous studies, the present study aims to further explore the role of Nrf2 and hepatic transporters in TP-induced hepatotoxicity and the protective effects of ISL by interfering with the endogenous system (BA transport) or directly altering the transport of xenobiotic toxins from the liver.

## Materials and Methods

### Chemicals and Reagents

Isoliquiritigenin (purity > 99%) and TP (purity ≥ 98%) were purchased from On-Road Biotechnology Co., Ltd. (Changsha, China); tBHQ, DMSO, MTT, E_2_17βG, GA, KCZ, BMR, and TBM were purchased from Sigma-Aldrich (St. Louis, MO, United States). TP, ISL, and tBHQ were dissolved in DMSO and stored at -20°C before use. The concentration of DMSO in the experiments never exceeded 0.1% (v/v). tBHQ, an Nrf2 inducer, was used as the positive control. CPA was obtained from the National Institute for Food and Drug Control (Beijing, China). NMQ was obtained from GemoMembrane (Yokohama, Japan). Nrf2-siRNA, negative control siRNA, and transfection reagents were obtained from Santa Cruz Biotechnology (Santa Cruz, CA, United States). Human MDR1 and human MRP2 vesicle products and VT assay reagent kits were from GemoMembrane (Yokohama, Japan). BAs were as follows: CA, DCA, CDCA, UDCA, HDCA, TCDCA, and TUDCA were from On-Road Biotechnology Co., Ltd. (Changsha, China); LCA, TCA, TDCA, TLCA, THDCA, GCA, GDCA, GLCA, GCDCA, and GUDCA were from Sigma-Aldrich (St. Louis, MO, United States). The antibodies included anti-Nrf2 (sc-722, Santa Cruz), anti-NQO1 (ab28947, Abcam), anti-P-gp (ab170904, Abcam), anti-MRP2 (ab203397, Abcam), anti-MRP4 (#12705, Cell Signaling Technology), anti-GAPDH (#MAB374, Merck Millipore), and anti-Lamin B2 (#12255, Cell Signaling Technology). All other reagents were of analytical reagent grade.

### Cell Culture

Human normal L02 hepatocytes obtained from the Cell Bank of the Chinese Academy of Sciences (Shanghai, China) were cultured in DMEM (Gibco, Grand Island, NY, United States) supplemented with 10% (v/v) FBS (BI, Israel) in a humidified incubator at 37°C with 5% CO_2_. The cells were treated with 50 nM TP for 24 h with or without 12 h ISL pretreatment. In all experiments, the cells were plated at an appropriate density according to the experimental design and grown for 24 h before the treatment.

For the cellular uptake assays, HEK293 cells stably expressing OATP1B1 (HEK293-OATP1B1), OATP1B3 (HEK293-OATP1B3), or respective vector control cells (HEK293-MOCK) were obtained from GemoMembrane (Yokohama, Japan) and cultured in DMEM (Gibco, Grand Island, NY, United States) supplemented with 10% (v/v) FBS (Gibco, Grand Island, NY, United States) and 1% antibiotics (100 μg/mL streptomycin/100 U/mL penicillin mix) (Gibco, Grand Island, NY, United States) in a humidified incubator at 37°C with 5% CO_2_.

### Cell Viability Assay

The cell viability was determined by MTT assay according to our previous studies (Cao et al., 2016a). L02 cells (2.5 × 10^4^ cells/well) were seeded in 24-well plates and treated with TP, ISL, or ISL + TP. Percent viability was defined as the relative absorbance of the treated cells versus the control cells. Morphological changes were detected with a light microscope (Nikon TS100, 10× magnification).

### Nrf2-siRNA Transient Transfection

L02 cells were transiently transfected with a mixture of transfection reagents and Nrf2-siRNA or negative control siRNA according to the manufacturer instructions. The cells were further treated as designed for cell viability assay 48 h after the transfection.

### Animal Treatments and Hepatotoxicity Assessments

All animal experiments were conducted according to the Regulations of Experimental Animal Administration issued by the State Committee of Science and Technology of China, with the approval of the Ethics Committee in the Experimental Animal Center of Hunan SJA Laboratory Animal Co. Ltd. (Changsha, China). All procedures were performed under urethane anesthesia, and all efforts were made to minimize the suffering.

Healthy male ICR mice, weighing 18–22 g, were provided by Hunan SJA Laboratory Animal Co. Ltd. (Changsha, China). The mice were kept at 22–25°C and humidity 50 ± 10% with a 12 h light-dark cycle and had free access to food and water. Thirty mice were randomly assigned to five groups (six in each group) and were respectively given the following treatments: (1) vehicle control, (2) TP (1.0 mg/kg), (3) ISL (25 mg/kg) + TP (1.0 mg/kg), (4) ISL (50 mg/kg) + TP (1.0 mg/kg), and (5) ISL (50 mg/kg). TP was injected intraperitoneally (i.p.), while ISL via oral gavage. The dosage of TP and ISL was decided based on our previous studies (Gong et al., 2015; Cao et al., 2016b). The mice received either 0.5% (w/v) CMC-Na or ISL once daily for 7 days consecutively. One hour after the final treatment, the mice were treated with TP (1.0 mg⋅kg^-1^, i.p.) or corresponding vehicles. Six hours after the TP administration, the mice were given either 0.5% (w/v) CMC-Na or ISL again. In all treatment groups, the mice were anesthetized 24 h after the TP injection. Blood samples were collected, and the liver of each mouse was collected and weighed. The degree of liver injury was assessed by H&E staining and serum biochemical parameters, including ALT, AST, ALP, and LDH and analyzed on an automatic clinical analyzer (7600, HITACHI Ltd., Tokyo, Japan).

### Quantitative Real-Time PCR

Total RNA was extracted with trizol reagent (Invitrogen Life Technologies, Carlsbad, CA, United States) according to the manufacturer’s instruction before being converted to cDNA with the PrimeScript^TM^ RT reagent Kit with gDNA Eraser (Takara, Japan). The cDNAs were analyzed with ABI Prism 7900HT (Applied Biosystems, United States) using an SYBR Premix Ex Taq (Takara, Japan). Each sample was run in duplicate and the results were normalized to the level of GAPDH mRNA. The primers were as follows: human MRP2 forward 5′-TGAGCAAGTTTGAAACGCACAT-3′ and reverse 5′-AGCTCTTCTCCTGCCGTCTCT-3′, mouse Bsep forward 5′-ACTCAGTGATTCTTCGCAGTGT-3′ and reverse 5′-CAAAGAAGCCAACTCGAGCG-3′, mouse Oatp2 forward 5′-TGATCGGACCAATCCTTGGC-3′ and reverse 5′-TCACAATGAAGCCGAGCCAC-3′, and the human and mouse GAPDH primers (Millipore, United States).

### Western Blot

The samples were lyzed with RIPA buffer (CW biotech, Beijing, China). Nuclear and cytoplasmic proteins were prepared with NE-PER nuclear and cytoplasmic extraction reagents (Pierce Biotechnology, Rockford, IL, United States) according to the manufacturer’s instruction. The same amounts of protein were loaded and separated by 10% SDS-PAGE electrophoresis and transferred to PVDF membranes. After being blocked in 5% nonfat milk in 0.05% Tween-20/tris-buffered saline for 1 h at room temperature, the membranes were incubated overnight at 4°C with relevant primary antibodies. The immunoblots were then incubated with species-specific secondary antibodies at room temperature. The membranes were developed with an ECL kit (Advansta, United States) according to manufacturer’s protocol.

### Vesicular Transport (VT) Assay

The VT assay was conducted as described in the VT Kit protocol (GenoMembrane, Yokohama, Japan). To determine whether TP inhibited the transport activities of P-gp and MRP2, human P-gp vesicles or human MRP2 vesicles were incubated with the test solution containing TP (0.01, 0.1, 1, 10, and 100 μM), the relative positive control, and the relative substrate with ATP or AMP at 37°C for 10 min. KCZ and BMR were used as positive inhibitors of P-gp and MRP2, respectively. NMQ, the substrate of P-gp, was analyzed by fluorescence measurement at λ_ex_ 355 nm and λ_em_ 460 nm (Molecular Devices i3-R, CA, United States). E_2_17βG, the substrate of MRP2, was analyzed with LC-MS/MS. To determine whether TP was a substrate of MRP2, human MRP2 vesicles were incubated with the test solution containing TP (0.01, 0.1, 1, 10, and 100 μM) or E_2_17βG serving as the positive control, with ATP or AMP at 37°C for 10 min. The vesicular accumulation of TP or E_2_17βG was measured by LC-MS/MS.

### Cellular Uptake Assay

To determine whether TP was a substrate of OATP1B1 or OATP1B3, HEK293-OATP1B1, HEK293-OATP1B3, and HEK293-MOCK cells were incubated with the test solution containing TP (0, 1, 10, and 100 μM) or E_2_17βG in the uptake buffer at 37°C for 10 min. Then the cells were washed three times with ice-cold uptake buffer and lyzed with the TBM solution to be used as the IS in the following analysis. The intracellular accumulation of TP or E_2_17βG was measured by LC-MS/MS, and the protein concentration of each sample was determined by bicinchoninic acid assay (Pierce BCA protein assay kit, Thermo Scientific, Rockford, IL, United States).

### LC-MS/MS for BAs, TP, and E_2_17βG

#### Sample Preparation

To analyze 16 BAs in the mouse liver, 0.1 g liver tissue was weighed and added to 800 μL 75% acetonitrile. After the homogenation, 100 μL homogenate was added into 300 μL IS solution. The mixture was vortexed for 3 min before centrifugation at 15000 rpm at 4°C for 10 min. Then the supernatant was collected and injected to the LC-MS/MS system.

To analyze TP or E_2_17βG in the vesicles, 50 μL 80% methanol was added to each well to dissolve the vesicles after washing and filtering. The obtained solution was centrifuged at 2000 rpm at 4°C for 2 min. These operations were repeated once. Then precooled methanol containing the relative IS was added to the supernatant. After centrifugation at 12000 rpm at 4°C for 5 min, the supernatant was collected and injected to the LC-MS/MS system.

To analyze TP in the HEK293 cells, precooled methanol containing the relative IS was added to the digested cells. After centrifugation at 12000 rpm at 4°C for 5 min, the supernatant was collected and injected to the LC-MS/MS system.

#### Instrumentation

An LC-20A HPLC system (SHIMADZU, Kyoto, Japan) coupled with a 4000 triple-quadrupole mass spectrometer (AB SCIEX, Framingham, MA, United States) was used to quantify BAs, TP, and E_2_17βG, respectively (Deng et al., 2015). For 16 BAs’ analysis, the separation was performed on an Xtimate^TM^ C_18_ column (2.1 mm × 150 mm, 3 μm, Welch, United States) analytical column connected with a top C_18_ column (Guard cartridge System, United States). The column temperature was 40°C. CPA (20 μg/mL) and GA (250 ng/mL) mixture solution was used as the IS. The gradient system consisted of solvent A (0.005% formic acid containing 7 mmol/L ammonium acetate) and solvent B (methanol) at a flow rate of 0.25 mL/min, and the gradient program was as follows: 60% B (0–2.0 min), 95% B (13.0–17.3 min), and 60% B (17.4–27.3 min). The mass spectrometer was used in MRM function in the ESI-negative mode. Other MS parameters were as follows: CUR 30.0 psi; medium CAD; ionspray voltage 5500.0 V; and ion source temperature 550.0°C.

For TP analysis, a Phenomenex Synergi Hydro RP column (2.0 mm × 30 mm, 4 μm, Phenomenex, United States) at 40°C was used for chromatographic separation. TBM was used as the IS. The gradient system consisted of solvent A (0.1% formic acid) and solvent B (methanol containing 0.1% formic acid) at 0.5 mL/min, and the gradient program was as follows: 30% B (0 min), 85% B (0.5–1.0 min), and 30% B (1.01–2.0 min). The mass spectrometer was used in MRM function in the ESI-positive mode. Other MS parameters were as follows: CUR 20.0 psi; CAD 6 psi; ionspray voltage 5000.0 V; and ion source temperature 500.0°C.

For E_2_17βG analysis, a Diamonsil C_18_ (2) column (4.6 mm × 50 mm, 5 μm, Diamonsil, United States) at 40°C was used for the chromatographic separation. TBM was used as the IS. The gradient system consisted of solvent A (0.05% ammonia) and solvent B (acetonitrile) at 0.85 mL/min, and the gradient program was as follows: 5% B (0 min), 70% B (2.0–2.5 min), and 5% B (2.51–3.3 min). The mass spectrometer was used in MRM function in the ESI-negative mode. Other MS parameters were as follows: CUR 20.0 psi; CAD 6 psi; ionspray voltage -4500.0 V; and ion source temperature 500.0°C.

### Statistical Analysis

The transport activity was expressed as pmol/min/mg protein. The ATP-dependent uptake of probe compound was calculated by subtracting the measured value of uptake in the absence of ATP from the value of uptake in the presence of ATP. The ATP-dependent transport of probe compound was set as 100%. To determine the effect of the test compounds, we calculated the ratio of the ATP-dependent uptake of probe compound in the absence and presence of the test compound resulting in normalized ATP-dependent transport (%). The UR in the VT assay was calculated by the transport activity in the presence of ATP dividing that in the presence of AMP, and that in the cellular uptake assay was calculated by the transport activity in OATP-expressing cells dividing that in the control cells. If the UR ≥ 2, the test drug is considered as a substrate of the transporter.

All experiments were conducted in duplicate. The data were presented as the means ± standard error (SE) or the means ± standard derivation (SD). Statistical analyses were performed with SPSS 20.0 for Windows (SPSS Inc., Chicago, IL, United States) or GraphPad Prism 5.0 software (GraphPad Software Inc., United States). One-way ANOVA followed by Dunnett’s *t*-test assessed the statistical significance of the difference between the mean values. A *P* ≤ 0.05 was considered significantly different.

## Results

### ISL Protected L02 Hepatocytes Against TP-Induced Cell Death

**FIGURE 1:**
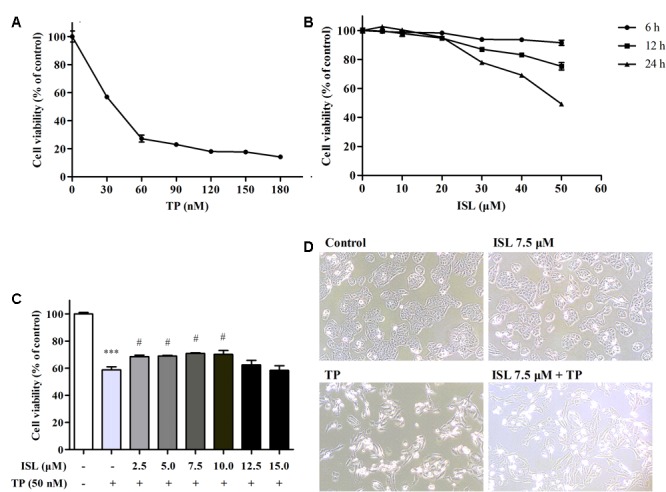
Effects of triptolide (TP), isoliquiritigenin (ISL), or ISL + TP on the viability and morphological changes of L02 cells. **(A)** L02 cells were treated with TP at various concentrations for 24 h. **(B)** L02 cells were treated with ISL at various concentrations for 6, 12, and 24 h. **(C)** L02 cells were pretreated with indicated ISL concentrations for 12 h followed by TP (50 nM, 24 h) treatment. **(D)** L02 cells were pretreated with 7.5 μM ISL for 12 h followed by TP (50 nM, 24 h) treatment. Cell morphological changes were detected by a light microscope (10 × magnification). The cell viability was determined by MTT assay. Data were presented as means ± standard error (*SE*) (*n* = 3); ^∗∗∗^*P* < 0.001 vs. the control group, ^#^*P* < 0.05 vs. the TP group, and ^###^*P* < 0.001 vs. the TP group.

Triptolide treatment alone for 24 h impaired the cell viability in a dose-dependent manner (**Figure 1A**). TP 50 nM was used in subsequent experiments, which was close to the IC50. ISL treatment alone showed no obvious cytotoxicity at 0–15 μM, but a time- and dose-dependent decrease of cell viability at 15–50 μM (**Figure 1B**). ISL pretreatment significantly attenuated the TP-induced reduction in cell viability at 2.5–10 μM. The protective effect was dose-dependent between 2.5 and 7.5 μM, and reached the maximum at 7.5 μM of ISL treatment (**Figure 1C**). Besides, TP treatment alone caused the cell detachment and distortion, while ISL pretreatment partially prevented cells from such changes (**Figure 1D**).

### Role of Nrf2 in ISL Protection Against TP-Induced Hepatotoxicity in L02 Cells

**FIGURE 2:**
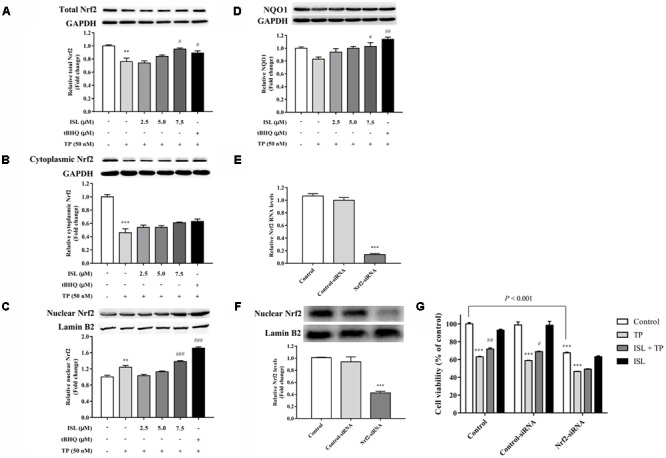
Role of nuclear transcription factor E2-related factor 2 (Nrf2) in TP-induced cell death and ISL-induced protective effects. Effects of TP, ISL, or ISL + TP on the protein levels of **(A)** total, **(B)** cytoplasmic, and **(C)** nuclear Nrf2 and **(D)** NAD(P)H: quinine oxidoreductase 1 (NQO1) in L02 cells. Cells were treated with 50 nM TP with or without 2.5, 5, and 7.5 μM ISL pretreatment. The tBHQ (15 μM) was used as an Nrf2 inducer. Cell lysates were analyzed by Western blot. Role of Nrf2 in the protection against TP-induced cytotoxicity in L02 cells. Cells were transiently transfected with Nrf2-siRNA or Control-siRNA before TP, ISL, or ISL + TP treatment. **(E)** RT-PCR analysis of Nrf2 mRNA levels 48 h after the transfection. **(F)** Western blot analysis of nuclear Nrf2 protein levels 72 h after the transfection. **(G)** Cell viability determined by MTT assay. Data were presented as means ±*SE* (*n* = 3); ^∗∗^*P* < 0.01 vs. the control group,^∗∗∗^*P* < 0.001 vs. the control group, ^#^*P* < 0.05 vs. the TP group, ^##^*P* < 0.01 vs. the TP group, and ^###^*P* < 0.001 vs. the TP group.

After confirming the effect of TP and ISL as well as the concentration and time of drug use, we further explored the mechanisms behind it. As shown in **Figures 2A–D**, protein levels of the total Nrf2, cytoplasmic Nrf2, and NQO1 decreased after the TP treatment, while the nuclear Nrf2 expression was induced. Compared with the TP-injured group, the total and nuclear Nrf2 and NQO1 protein levels were significantly increased after ISL (7.5 μM) or tBHQ (15 μM) pretreatment.

To further determine whether the ISL protection against TP-induced L02 injuries was Nrf2-dependent, Nrf2-siRNA was transfected into L02 cells. Nrf2-siRNA transfection led to a significant knockdown of Nrf2 at both mRNA and protein levels, while negative control siRNA had no effect on Nrf2 expressions (**Figures 2E,F**). Nrf2 knockdown significantly reduced the cell viabilities, and ISL treatment did not significantly protect Nrf2-siRNA transfected cells from TP-induced injuries (**Figure 2G**).

### Effect of TP and ISL Treatment on Expressions of MRP2, MRP4, and P-gp in L02 Cells

**FIGURE 3:**
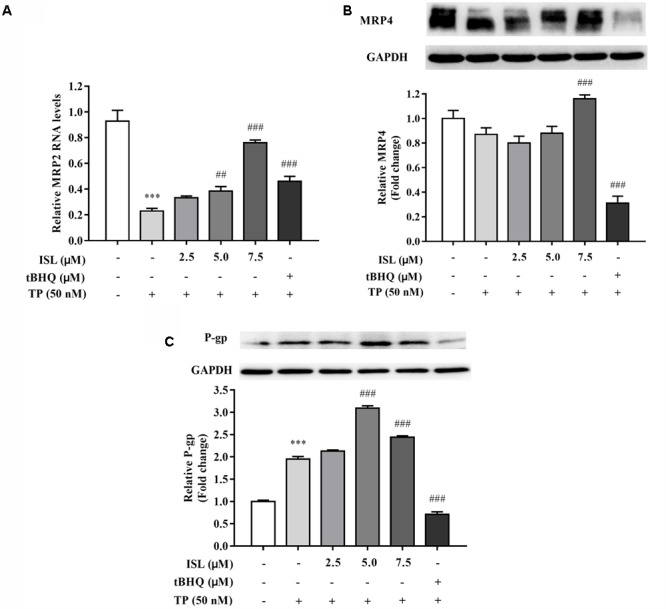
ISL pretreatment induced expressions of **(A)** MRP2, **(B)** MRP4, and **(C)** P-gp to protect against TP-induced cytotoxicity. L02 cells were treated with 50 nM TP with or without 2.5, 5, and 7.5 μM ISL pretreatment. The *tert*-butylhydroquinone (15 μM) was used as an Nrf2 inducer. RT-PCR was used to analyze mRNAs and proteins by Western blot. Data were presented as means ±*SE* (*n* = 3);^∗∗∗^*P* < 0.001 vs. the control group, ^##^*P* < 0.01 vs. the TP group, and ^###^*P* < 0.001 vs. the TP group.

We detected the changes in the expression levels of Nrf2-regulated and -nonregulated transporters after the TP and ISL treatment. MRP2 mRNA levels of TP-injured groups significantly decreased, and decrease in MRP4 protein levels was insignificant, while ISL pretreatment increased MRP2 mRNA levels in a concentration-dependent manner (**Figures 3A,B**). ISL pretreatment significantly increased MRP4 protein levels at 7.5 μM, but tBHQ had no induction effect on MRP4. TP treatment induced P-gp expressions in L02 cells, and ISL pretreatment intensified this induction (**Figure 3C**).

### Protective Effect of ISL on TP-Induced Liver Injury in Mice

The results of *in vitro* studies were validated *in vivo*. The liver indexes, histopathology, and serum biochemical indices were analyzed to determine the degree of liver injury. The liver index significantly increased in the TP-injured group, while ISL combined treatment decreased the liver index though the result was not statistically significant (**Table 1**).

**Table 1:** Effects of triptolide (TP), isoliquiritigenin (ISL), or ISL + TP on the liver indexes [means ± standard deviation (*SD*), *n* = 5–6].

Group	Liver weight (g)	Weight (g)	Liver index (%)
Control	1.28 ± 0.15	26.32 ± 1.53	4.87 ± 0.31
TP	1.69 ± 0.12	27.16 ± 1.04	6.23 ± 0.26^∗∗∗^
25 mg/kg ISL + TP	1.53 ± 0.06	26.25 ± 1.94	5.85 ± 0.41
50 mg/kg ISL + TP	1.62 ± 0.12	27.58 ± 1.30	5.87 ± 0.22
50 mg/kg ISL	1.27 ± 0.12	26.87 ± 1.70	4.73 ± 0.30

*^∗∗∗^*P* < 0.0001 vs. the control group.*

In the TP-injured group, the serum activities of ALT, AST, and LDH significantly increased, and combined ISL treatment significantly reduced the serum ALT and AST activities (**Table 2**).

**Table 2:** Blood chemistry of male ICR mice administered TP, ISL, or both (mean ± SD, *n* = 5–6).

Group	ALT (U/L)	AST (U/L)	ALP (U/L)	LDH (U/L)
Control	30.23 ± 1.83	95.28 ± 13.15	128.00 ± 11.01	1091.96 ± 122.93
TP	59.73 ± 31.65^∗^	214.30 ± 81.68^∗∗∗^	113.23 ± 14.43	1567.90 ± 330.94^∗∗^
25 mg/kg ISL + TP	32.87 ± 3.45^#^	166.33 ± 17.57	110.07 ± 26.02	1658.67.07 ± 203.40
50 mg/kg ISL + TP	28.97 ± 1.30^#^	144.10 ± 8.24^#^	103.66 ± 12.77	1393.36 ± 143.62
50 mg/kg ISL	30.17 ± 4.48	121.70 ± 26.35	147.63 ± 11.58	1255.03 ± 120.71

*ALT, alanine aminotransferase; AST, aspartate aminotransferase; ALP, alkaline phosphatase; LDH, lactate dehydrogenase. ^∗^*P* < 0.05 vs. the control group,^∗∗^*P* < 0.001 vs. the control group, ^∗∗∗^*P* < 0.0001 vs. the control group, and ^#^*P* < 0.05 vs. the TP group.*

**FIGURE 4:**
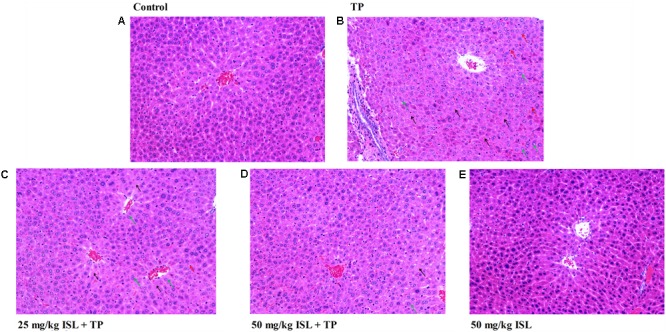
Photomicrographs (× 200) of hematoxylin & eosin-stained liver sections obtained from the **(A)** control, **(B)** TP (1.0 mg/kg), **(C)** ISL (25 mg/kg) + TP (1.0 mg/kg), **(D)** ISL (50 mg/kg) + TP (1.0 mg/kg), and **(E)** ISL (50 mg/kg) groups. Green arrows indicate hepatocellular hydropic degeneration, black arrows indicate necrosis, and red arrows indicate inflammatory cell infiltration.

Histopathological analysis showed that the liver sections of the TP group showed a derangement of the hepatic cord and large areas of hydropic degeneration, compared with those of the control group. Nucleolysis and further hepatic parenchymal necrosis and some inflammatory cells infiltration were also seen in the TP group, but not in the positive control group. The severity of histopathological lesion was significantly decreased in the ISL + TP treatment group, showing less hepatocyte degeneration, and the hydropic degeneration was mostly near the central vein (**Figure 4**).

### Role of Nrf2 in ISL Protection Against TP-Induced Hepatotoxicity in Mice

**FIGURE 5:**
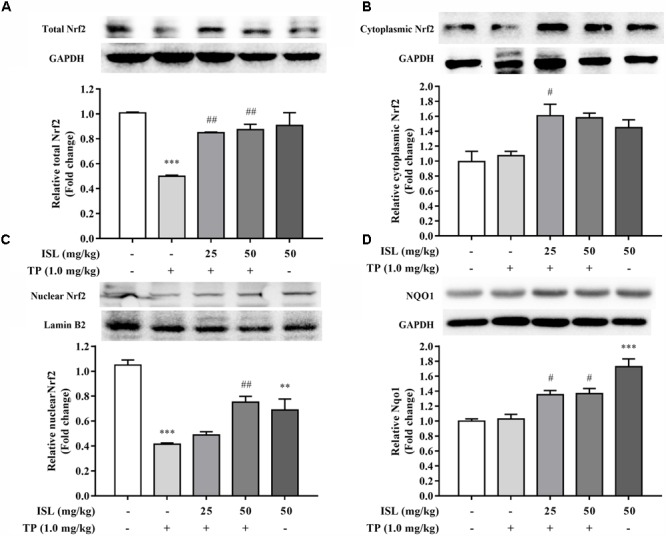
Effects of TP, ISL, or ISL + TP on protein levels of **(A)** total, **(B)** cytoplasmic, and **(C)** nuclear Nrf2 and **(D)** Nqo1 in mouse livers. Cell lysates were analyzed by Western blot. Data were presented as means ±*SE* (*n* = 5); ^∗∗^*P* < 0.01 vs. the control group,^∗∗∗^*P* < 0.001 vs. the control group, ^#^*P* < 0.05 vs. the TP group, and ^##^*P* < 0.01 vs. the TP group.

The total, cytoplasmic, and nuclear Nrf2 expressions were also detected in the mouse livers. As shown in **Figure 5**, TP administration reduced the total and nuclear levels of Nrf2, while combined ISL treatment induced the expressions of total, cytoplasmic, and nuclear Nrf2 as well as its downstream Nqo1.

### Effect of TP and ISL Administration on the Expression of Hepatic Transporters in Mice

**FIGURE 6:**
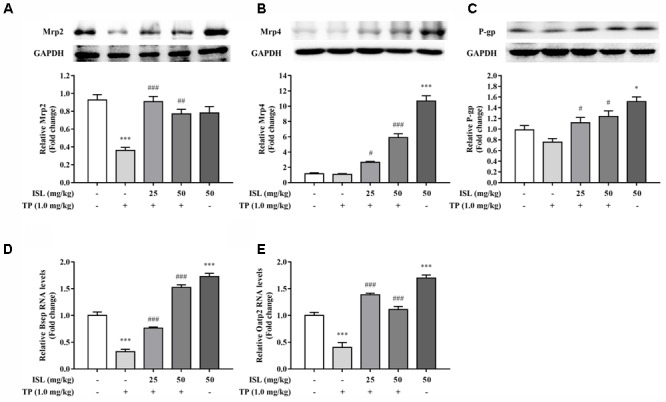
Effects of TP, ISL, or ISL + TP on the protein levels of **(A)** MRP2, **(B)** MRP4, and **(C)** P-gp and the mRNA levels of **(D)** Bsep and **(E)** Oatp2 in mouse livers. Cell lysates were analyzed by Western blot and mRNAs by RT-PCR. Data were presented as means ±*SE* (*n* = 5); ^∗^*P* < 0.05 vs. the control group,^∗∗∗^*P* < 0.001 vs. the control group, ^#^*P* < 0.05 vs. the TP group, and ^###^*P* < 0.001 vs. the TP group.

Protein levels of Mrp2 decreased in the TP-injured group, and combined ISL treatment caused a dramatic increase in the Mrp2 expression (**Figure 6A**). Combined ISL treatment also dose-dependently increased the protein levels of Mrp4 and P-gp (**Figures 6B,C**).

Significant decrease in the mRNA levels of Bsep and Oatp2 was found in the TP-injured group, and combined ISL treatment resulted in an increase in the expressions of Bsep and Oatp2 (**Figures 6D,E**).

### Changes of Hepatic Bile Acid Profiles in Mouse Livers

**FIGURE 7:**
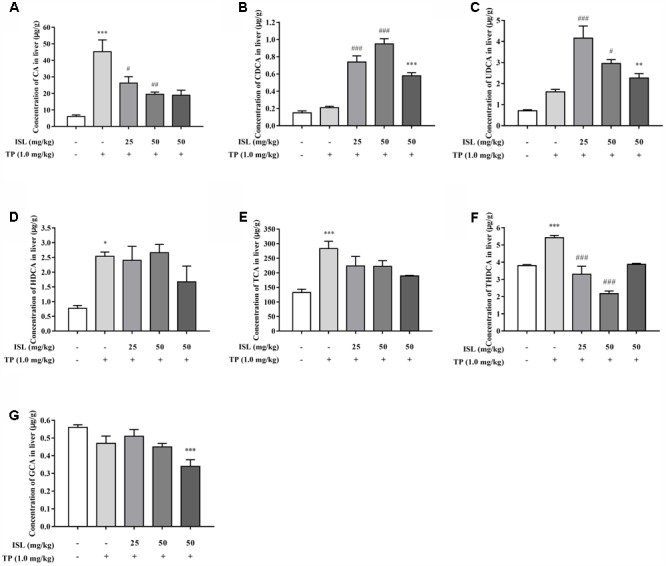
Effects of TP, ISL, or ISL + TP on the hepatic bile acid (BA) profiles. Significant changes of hepatic concentration after TP or ISL administration were found in **(A)** CA, **(B)** CDCA, **(C)** UDCA, **(D)** HDCA, **(E)** TCA, **(F)** THDCA and **(G)** GCA. Concentrations of 16 BAs in mouse livers were quantified by liquid chromatography-tandem mass spectrometry. Data were presented as means ± SE (*n* = 4–6); ^∗^*P* < 0.05 vs. the control group, ^∗∗^*P* < 0.01 vs. the control group, ^∗∗∗^*P* < 0.001 vs. the control group, ^#^*P* < 0.05 vs. the TP group, ^##^*P* < 0.01 vs. the TP group, and ^###^*P* < 0.001 vs. the TP group.

To further validate the role of transporters, the hepatic concentrations of 16 BAs were analyzed. Concentrations of LCA, GDCA, GCDCA, and GUDCA were under the lower limit of detections. TP administration dramatically increased the hepatic concentrations of CA, HDCA, TCA, and THDCA, compared with the control group, while the hepatic CA and THDCA, accumulation significantly reduced in ISL combined treatment (**Figure 7**). Besides, ISL combined treatment increased the concentrations of CDCA and UDCA in the mouse livers compared with those of the TP-treatment group.

### TP Did Not Inhibit the Transport Activities of P-gp and MRP2

**FIGURE 8:**
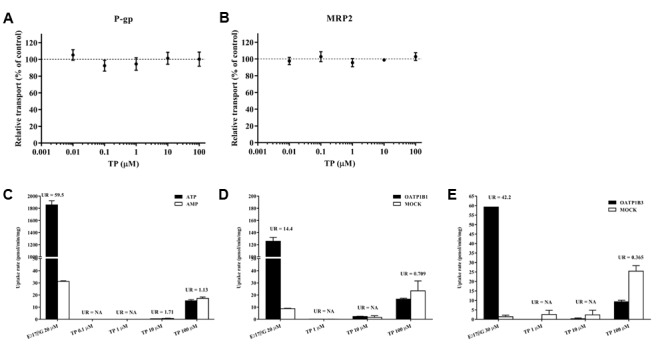
*In vitro* transport assay to determine the interaction between TP and hepatic transporters. **(A,B)** Effects of TP on P-gp and MRP2-mediated transports in the vesicular transport (VT) assay. TP was incubated with relative membrane vesicles and substrates at 37°C for 10 min. N-methyl quinidine was used as the positive control. The ATP-dependent transport in the absence of TP was set as 100%. **(C–E)** Uptake of TP by MRP2 in the VT assay as well as OATP1B1 and OATP1B3 in human embryonic kidney 293 cells. Membrane vesicles or cells were incubated with TP at 37°C for 10 min. E_2_17βG was used as the positive control. Uptake ratio (UR) in the MRP2 vesicles was calculated by the transport activity in the presence of ATP dividing that in the presence of AMP. UR in the OATP-expressing cells was calculated by the transport activity in OATP-expressing cells dividing that in the control cells. Data were presented as means ±*SE* (*n* = 3).

After determining changes in the transporter expressions, we further investigated whether TP inhibited the activity of human P-gp or MRP2. The UR of the substrate in the VT systems was 3.31 and 59.53 for P-gp and MRP2 vesicles, both greater than 2, indicating that the VT systems were running well. The relative transport of NMQ in the positive control group was 11.05% ± 2.53% for P-gp and 12.80% ± 0.81% for MRP2 vesicles. The relative transport of the substrates was not influenced by TP co-incubation, indicating that TP had no effect on the P-gp or MRP2 mediate transport in the vesicles (**Figures 8A,B**).

### TP Was Not Transported by MRP2, OATP1B1, or OATP1B3

The UR of E_2_17βG, a substrate of MRP2, OATP1B1, and OATP1B3 which served as the positive control, was more than 2. TP concentrations in the vesicles or cells were under the lower limit of quantification when the incubation TP concentration was 0.1, 1, and 10 μM, respectively. The UR of 100 μM TP was less than 2 in the MRP2, OATP1B1, and OATP1B3 transport systems, indicating that TP was not a substrate of MRP2, OATP1B1, or OATP1B3 (**Figures 8C–E**).

## Discussion

Since TP-induced liver injury is always acute clinically (Xu et al., 2013), in this study, we established acute hepatotoxicity models by single-dose TP treatment in both L02 cells and ICR mice. Our data showed that TP treatment induced hepatic damage both *in vitro* and *in vivo*, indicating successful modeling and relieving effect of ISL combined treatment, which is consistent with our previous studies (Cao et al., 2016a,b). The present study for the first time used human normal L02 hepatocytes rather than hepatoma cells or rodent hepatocytes, which is as close to clinical practice as possible, since there are differences in physiological conditions as well as distributions and expression levels of transporters between human normal hepatocytes and hepatoma cells or rodent hepatocytes (Nicolaou et al., 2012; Le Vee et al., 2013).

As is known to all, antioxidants may react with MTT reagents and interfere with the results. However, in the present study, the ISL solution was removed and the cells were washed with PBS twice before adding MTT to each well. To determine the protective effect of ISL on TP-induced cell death, the cells were preincubated with ISL, then ISL was removed and TP was added. Hence, ISL had no direct contact with MTT during the whole study, so it did not influence the results of the MTT assay.

Alanine aminotransferase is mainly distributed in the liver, whose increase is a sensitive marker of acute liver injury. AST is mainly distributed in the heart and followed by the liver. Obviously bigger increase in the AST than in the ALT is observed in the following conditions:

(1) When cardiomyocyte injuries appear, such as TP-induced cardiotoxicity (Yang et al., 2016).
(2) When prejaundill occurs. Clinical symptoms of TP-induced liver injuries include jaundice (Xue et al., 2009), and Zhang et al. (2018) found that *tripterygium wilfordii* significantly increased the total bilirubin in rat serum, which may be related to the changes in transporter expressions.
(3) When the mitochondria are injured, a large amount of AST may leak into the serum, so the big increase in the AST may be a sign of serious liver injury.

In addition, the serum level of LDH is a sign of cell necrosis or apoptosis, during which LDH may leak out from the cells since the membrane is broken (Zhou and Ho, 2014). The serum activities of LDH were not reduced after the combined ISL treatment compared with those of the TP-injured group, and it was not obviously increased, suggesting the cytotoxic effect of ISL. However, LDH is widely distributed in all organs, so increase in the serum LDH levels does not necessarily mean ISL induced liver injuries; it is possible that ISL may have side effect on other organs. Unfortunately, there are very few toxicological evaluations reported on ISL. Mahalingam et al. (2016) proved that ISL inhibited the growth of antral follicle and sex steroid synthesis in adult mouse ovaries. Several studies investigated the anticancer effect of ISL and showed that ISL could kill cancer cells by inducing the apoptotic pathways or disturbance of redox status (Sun et al., 2013; Zhou and Ho, 2014). It was reported that the decreased ROS caused by ISL might lead to redox imbalance and reductive stress. To adapt to this state, Nrf2 could be significantly decreased. Thus, ISL may induce the oxidative stress by disturbing the redox status (Sun et al., 2013). That may be the reason why low-dose ISL did not induce nuclear Nrf2 in L02 cells and why the serum levels of LDH was not reduced compared with those of the TP-injured group with ISL combined treatment. Further studies are needed to verify the target-organ toxicity or side effects (Peng et al., 2015). Anyway, the hepatoprotective effect of ISL is definite and outstanding (Kuang et al., 2017), and the levels of ALT and AST, the two sensitive markers of acute liver injuries, are significantly reversed by ISL combined treatment, implying the hepatoprotective effect of ISL.

We try to explain the mechanisms behind it. Our previous studies have explored the total Nrf2 expression levels in HepG2 cells and mouse livers after TP and ISL treatment (Cao et al., 2016a,b), while as a nuclear transcription factor, Nrf2 was translocated into the nucleus to activate the target protective gene transcription (Suzuki et al., 2013). The present study determined the total, cytoplasmic, and nuclear Nrf2 protein levels as well as its downstream antioxidant NQO1, which is regarded as a prototypical Nrf2-target gene whose induction implies the activation of a host of other Nrf2-regulated cytoprotective genes (Yates et al., 2007). Our results showed that compared with the TP-injured group, ISL pretreatment significantly enhanced the total and nuclear Nrf2 as well as NQO1 expressions *in vitro* and *in vivo*, indicating that Nrf2 is more stabilized with ISL combined treatment, accumulated into the nucleus, and has activated its target protective genes. The increased expression of cytoplasmic Nrf2 is not statistically significant. Since Nrf2 is translocated into the nucleus and plays a protective role, the increased nuclear translocation of Nrf2 may lead to a relative decrease in the cytoplasmic Nrf2 expression level. When Nrf2 was knocked down by siRNA, TP-induced cytotoxicity in the L02 cells was enhanced, and the protective effect of ISL was diminished, further proving the role of Nrf2 in TP-induced hepatotoxicity and the protective effect of ISL. However, the Nrf2 influence was relatively low. **Figure 2G** shows that in the control group, ISL pretreatment improved the cell viability by 8.8% compared with that of the TP-injured group, while the cell viability in the Nrf2-siRNA group improved by 2.8%. Although ISL pretreatment still had some recovering effect on the cell viabilities after Nrf2-siRNA transfections, this protection diminished a lot. The remaining protective effect may be due to other nuclear factors and non-Nrf2-regulated transporters. Our present study also investigated the role of P-gp and OATPs in the protective effect of ISL on TP-induced hepatotoxicity. P-gp exports TP from the hepatocytes, and OATPs import BAs into the hepatocytes. Alterations of these two transporter expressions by ISL lead to low concentrations of TP and BAs in the liver, which is also an efficient way to alleviate TP-induced hepatotoxicity. Besides, increased nuclear Nrf2 level in the TP-injured group *in vitro* is an adaptive activation to alleviate or delay the TP-induced injury, since it was unable to completely eliminate the toxicity which was related to the time or dosage of TP treatment (Li J. et al., 2014; Xi et al., 2017; Zhou et al., 2017).

Considering that Nrf2 is the primary player in the inducible cell defense system, much interest is shown in identifying and developing Nrf2 activators for therapeutic use (Suzuki et al., 2013). Some of the most promising Nrf2 inducers include SFN, oltipraz, BHA, natural triterpenoid oleanolic acid, and synthetic triterpenoids (CDDO, CDDO-Me, and CDDO-Im), all of which have been shown to be able to protect the liver in different models of oxidative and electrophilic stress by activating the Nrf2 expressions (Klaassen and Reisman, 2010). As a flavonoid contains multiple phenolic hydroxyl groups, ISL also shows great antioxidant effect (Chin et al., 2007). Our previous studies show that ISL is the most potent ARE-luciferase inducer among the four main components of licorice, and it may increase the Nrf2 protein level and nuclear accumulation. Park et al. (2016) have also proved that ISL can function as a hepatic protectant by inducing the antioxidant genes through extracellular signal-regulated kinase-mediated Nrf2 pathway both *in vitro* and *in vivo*. Therefore, ISL may be a novel Nrf2-activating drug with hepatoprotective effect, but the molecular mechanisms through which ISL counteracts with Nrf2 need further clarification. In the present study, induction of nuclear Nrf2 by ISL does not imply oxidative stress; in contrast, it implies antioxidant effects on TP-induced hepatotoxicity. Moreover, the reduction of ROS and induction of GSH in our preliminary study also proved its protective effect (Cao et al., 2016a). Clearly, duration of exposure and dose selection are critical parameters to assess safe levels of TP and ISL. Further studies are needed to conduct an ISL risk assessment by evaluating three main data sets: animal toxicology data, human intervention studies, and published case reports and publicly available adverse event reports, to get the optimal exposure time and dose and to prevent the adverse effect of ISL (Yates et al., 2017). As a key regulator of detoxification and BA metabolism, Nrf2 induces a series of hepatic efflux transporters that aid in the elimination of potentially harmful endo- and xenobiotics (Klaassen and Reisman, 2010; Zhang et al., 2017). Since TP-induced hepatotxicity is correlated with its hepatic exposure and BA accumulation which may lead to cholestatic symptoms (Kong et al., 2015; Jiang et al., 2016; Yang et al., 2017), and licorice and its main constitutes have been proved to induce the expressions of an array of Nrf2 downstream transporters and inhibit chelostasis (Wang X. et al., 2013; Gong et al., 2015), we measured changes in the expression of hepatic transporters after TP and ISL treatment. TP treatment reduced the expressions of MRP2 *in vitro* and *in vivo*, while ISL combined treatment reversed this effect. MRP2 is an Nrf2 downstream transporter that locates in the canalicular membrane and exports many substrates including multiple drugs and its phase II conjugates as well as BAs (Csanaky et al., 2009; Klaassen and Reisman, 2010). It plays a major role in the formation of BA-independent bile flow, which is important for excretion of endobiotic and xenobiotic toxins (Klaassen and Aleksunes, 2010; Nicolaou et al., 2012). Hence, alterations in the expression of MRP2 may get involved in TP-induced hepatotoxicity and ISL-induced protective effect, resulting in changes in the hepatic accumulation of potential toxins. Compared with the TP-injured group, ISL combined treatment also increased the protein level of MRP4, a transporter that exports BAs and drugs/metabolites across the hepatic basolateral membranes, resulting in a further elimination of toxins from the liver (Hillgren et al., 2013). In addition, decrease in MRP4 expression level in L02 cells in the TP-injured group was not statistically significant, which might be the result of a compensatory role of MRP4 when MRP2 was impaired (Hillgren et al., 2013). Notably, pretreatment with tBHQ, an Nrf2 inducer, did not increase the MRP4 protein level *in vitro*, which may be attributed to the fact that besides Nrf2, many other nuclear factors are also involved in the regulation of MRP4 expressions, especially the CAR as a key regulator (Zollner et al., 2010). Considering that the Nrf2-regulated Bsep mediates the rate-limiting step of BA transport across hepatocytes (Nicolaou et al., 2012; Hillgren et al., 2013), we also measured the expression of Bsep *in vivo*. TP administration reduced the Bsep mRNA levels, implying a disruption of BA homeostasis, while combined ISL treatment improved the Bsep mRNA levels and led to a protective effect. In addition, since TP is a substrate of P-gp (Zhuang et al., 2013), the protein level of P-gp has also been measured, showing that ISL combined treatment induced P-gp expressions, which may help eliminate TP from the liver into the bile. Apart from the hepatic efflux transporters, we also detected the mRNA level of mouse Oatp2, a hepatic influx transporter with a broad substrate specificity, involved in the enterohepatic recycling of BAs (Obaidat et al., 2012). We found that TP administration reduced Oatp2 expression while ISL combined treatment induced it, suggesting a role of Oatp2 in TP-induced hepatotoxicity and the protective effect of ISL.

**FIGURE 9:**
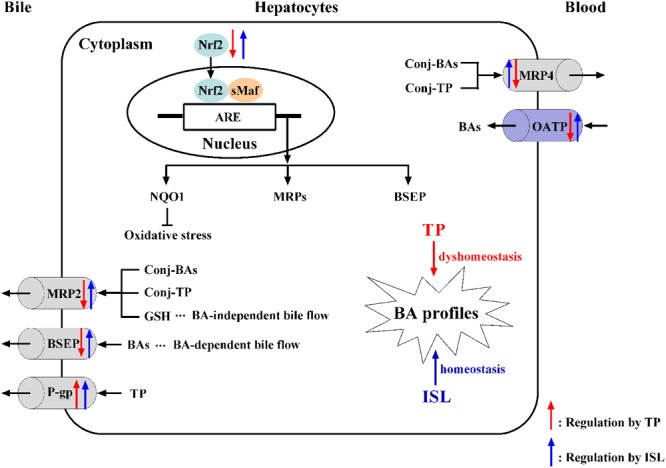
The proposed mechanism of TP-induced hepatotoxicity and ISL combined treatment protecting against TP-induced liver injury. ARE, antioxidant responsive element; BA, bile acid; BSEP, bile salt export pump; conj, conjugated; GSH, reduced glutathione; ISL, isoliquiritigenin; MRP, multidrug resistance protein; NQO1, NAD(P)H: quinine oxidoreductase 1; Nrf2, nuclear transcription factor E2-related factor 2; OATP, organic anion transporting polypeptide; P-gp, P-glycoprotein; sMaf, small Maf proteins; TP, triptolide.

Given the alterations in the expressions of hepatic transporters and the reported results that TP administration increased TBA accumulation in rat livers (Jiang et al., 2016; Yang et al., 2017), we further validated whether changes in the expressions of transporters have any effect on hepatic BA accumulation by analyzing the profile of individual BAs in mouse livers with our previously established LC-MS/MS method. The results showed that TP induced 4 out of the 16 BAs, which may result in a cholestatic phenotype, while ISL combined treatment decreased the hepatic accumulation of CA and THDCA, which relieved TP-induced BA disorder. What is more, ISL combined treatment increased the hepatic concentration of CDCA and UDCA. Increase in UDCA might be cytoprotective by lowering the intracellular TCDCA, which is thought to be cytotoxic due to its hydrophilic nature (Guo et al., 2016). It has been reported that CDCA and UDCA can alleviate 17α-ethinylestradiol-induced cholestasis in rats, and UDCA can stimulate biliary secretion, upregulate the expression of Bsep and Mrp2, and stabilize Bsep protein in the apical membrane (Li et al., 2016). Hence, the increase in CDCA and UDCA levels induced by ISL may also have a protective effect on the TP-induced liver injury. The proposed mechanism is shown in **Figure 9**.

Above we have validated that TP and ISL treatment changed the expression of hepatic transporters. Since changes in transporter activities may also contribute to various drug and BA exposure and response, we tried to determine whether TP inhibited the activities of human P-gp and MRP2, or whether TP interacted with them. The reason for choosing these two transporters is that P-gp exports TP, and MRP2 has a wide range of substrates and its expression is inhibited by TP, so inhibition of P-gp and MRP2 transport may lead to the accumulation of TP or other substrates and finally result in hepatotoxicity. The VT assay utilizing inverted membrane vesicles enables direct interaction of a transporter with the compounds added to the reaction buffer. Therefore, it is a high-throughput *in vitro* method to identify the substrates, inhibitors, or modulators of transporters (Kidron et al., 2012), and can also avoid the compensatory effects between transporters. Our results showed that TP was not an inhibitor of P-gp or MRP2, suggesting that the effect of TP on transporters is at transcription levels, which is usually more powerful than that at functional levels (Kong et al., 2015).

Furthermore, we explored whether TP could be transported by several hepatic transporters and contribute to the hepatic TP accumulation. We found that TP was not a substrate of MRP2, OATP1B1, or OATP1B3, suggesting that these transporters mainly contributed to the hepatic accumulation of BAs but not TP. Besides, phase II metabolites of TP rather than the parent drug may be transported by MRP2, which may also contribute to the TP-induced hepatotoxicity.

## Conclusion

Nrf2 and hepatic influx and efflux transporters play important roles in TP-induced hepatotoxicity and ISL-induced protective effect by modulating the hepatic BA profiles as well as the accumulation of TP and its metabolites. We also confirm that TP is not the substrate of MRP2, OATP1B1, or OATP1B3, nor does it inhibit the transport activity of MRP2 or P-gp, thus shedding new lights on TP-related drug–drug interactions as well as the mechanisms of TP-induced hepatotoxicity.

## Author Contributions

ZH, BZ, and MY designed the experiments. ZH, LeC, PF, HC, HT, and YP conducted the experiments. ZH, LeC, YD, and LiC analyzed the data. ZH, BZ, and MY wrote the paper. HL proposed the research idea. All authors contributed to the editing of the paper and to scientific discussions.

## Conflict of Interest Statement

The authors declare that the research was conducted in the absence of any commercial or financial relationships that could be construed as a potential conflict of interest.

## Abbreviations

ALP: alkaline phosphatase
ALT: alanine aminotransferase
ANOVA: analysis of variance
ARE: antioxidant responsive element
AST: aspartate aminotransferase
BA: bile acid
BCRP: breast cancer resistance protein
BMR: benzbromarone
BSEP: bile salt export pump
CA: cholic acid
CAD: collision gas
CAR: constitutive androstane receptor
CDCA: chenodeoxycholic acid
CPA: cyproterone acetate
CUR: curtain gas
CYP3A: cytochrome P450 3A
DCA: deoxycholic acid
DILI: drug-induced liver injury
DMEM: Dulbecco Modified Eagle Medium
DMSO: dimethyl sulfoxide
E_2_17βG: estradiol-17-β-D-glucuronide
ECL: electrochemiluminescence
ESI: electrospray ionization
FBS: fetal bovine serum
FXR: farnesoid X receptor
GA: glycyrrhetinic acid
GCA: glycocholic acid
GCDCA: glycochenodeoxycholic acid
GDCA: glycodeoxycholic acid
GLCA: glycolithocholic acid
GSH: reduced glutathione
GUDCA: glycoursodeoxycholic acid
H&E: hematoxylin & eosin
HDCA: hyodeoxycholic acid
HEK293: human embryonic kidney 293
IC50: half maximal inhibitory concentration
IS: internal standard
ISL: isoliquiritigenin
KCZ: ketoconazole
LC-MS/MS: liquid chromatography-tandem mass spectrometry
LCA: lithocholic acid
LDH: lactate dehydrogenase
MRM: multiple reaction monitoring
MRP: multidrug resistance protein
MTT: 3-(4, 5-dimethylthiazol-2-yl)-2; 5-diphenyltetrazolium bromide
NA: not applicable
NMQ: N-methyl quinidine
NQO1: NAD(P)H: quinine oxidoreductase 1
Nrf2: nuclear transcription factor E2-related factor 2
OATP: organic anion transporting polypeptide
P-gp: P-glycoprotein
*SD*: standard deviation
tBHQ: *tert*-butylhydroquinone
TBM: tolbutamide
TCA: taurocholic acid
TCDCA: taurochemodeoxycholic acid
TDCA: taurodeoxycholic acid
THDCA: taurohyodeoxycholic acid
TLCA: taurolithocholic acid
TP: triptolide
TUDCA: tauroursodeoxycholic acid
TwHF: *Tripterygium wilfordii* Hook F
UDCA: ursodeoxycholic acid
UR: uptake ratio
VT: vesicular transport
